# *catena*-Poly[[[tetra­aqua­copper(II)]-μ-4,4′-bipyridyl-κ^2^ *N*:*N*′] tetra­fluorido­succinate tetra­hydrate]

**DOI:** 10.1107/S1600536812014948

**Published:** 2012-04-18

**Authors:** Guo-Jun Yu, Lan-Ping Xu, Lan Qin, Lei Han

**Affiliations:** a Faculty of Materials Science & Chemical Engineering, Ningbo University, Ningbo, Zhejiang 315211, People’s Republic of China

## Abstract

In the title compound, {[Cu(C_10_H_8_N_2_)(H_2_O)_4_](C_4_F_4_O_4_)·4H_2_O}_*n*_, the Cu^II^ atom adopts an elongated octa­hedral geometry because of the Jahn–Teller effect. Both cation and anion have crystallographic twofold rotation symmetry with the twofold axes passing through the Cu and N atoms and through the midpoint of the central C—C bond. The 4,4′-bipyridyl ligand links the Cu^II^ atoms into a linear chain along the *b* axis. O—H⋯O hydrogen-bonding inter­actions between the cationic chains and the tetra­fluorido­succinate anions and the free water mol­ecules generate a three-dimensional supra­molecular network.

## Related literature

For background to metal-organic framework structures, see: Allendorf *et al.* (2009 ▶). For the construction of hybrid frameworks with perfluorinated ligands, see: Yang *et al.* (2007 ▶); Hulvey *et al.* (2009 ▶).
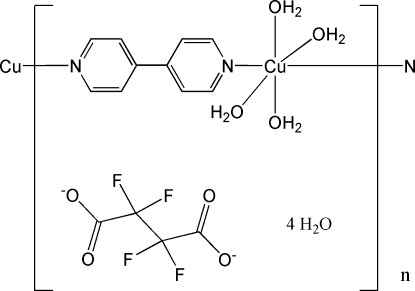


## Experimental

### 

#### Crystal data

[Cu(C_10_H_8_N_2_)(H_2_O)_4_](C_4_F_4_O_4_)·4H_2_O
*M* *_r_* = 551.89
Monoclinic, 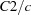

*a* = 17.112 (3) Å
*b* = 11.135 (2) Å
*c* = 12.126 (2) Å
β = 104.85 (3)°
*V* = 2233.3 (7) Å^3^
*Z* = 4
Mo *K*α radiation
μ = 1.07 mm^−1^
*T* = 298 K
0.44 × 0.22 × 0.10 mm

#### Data collection

Bruker SMART APEX diffractometer
Absorption correction: multi-scan (*SADABS*; Bruker, 2005 ▶) *T*_min_ = 0.650, *T*_max_ = 0.900
10662 measured reflections
2546 independent reflections
2115 reflections with *I* > 2σ(*I*)
*R*_int_ = 0.026

#### Refinement

*R*[*F* ^2^ > 2σ(*F* ^2^)] = 0.027
*wR*(*F* ^2^) = 0.085
*S* = 1.28
2546 reflections
153 parameters
H-atom parameters constrained
Δρ_max_ = 0.70 e Å^−3^
Δρ_min_ = −0.78 e Å^−3^

### 

Data collection: *SMART* (Bruker, 2005 ▶); cell refinement: *SAINT* (Bruker, 2005 ▶); data reduction: *SAINT*; program(s) used to solve structure: *SHELXS97* (Sheldrick, 2008 ▶); program(s) used to refine structure: *SHELXL97* (Sheldrick, 2008 ▶); molecular graphics: *SHELXTL* (Sheldrick, 2008 ▶); software used to prepare material for publication: *SHELXTL*.

## Supplementary Material

Crystal structure: contains datablock(s) I, global. DOI: 10.1107/S1600536812014948/mw2062sup1.cif

Structure factors: contains datablock(s) I. DOI: 10.1107/S1600536812014948/mw2062Isup2.hkl

Additional supplementary materials:  crystallographic information; 3D view; checkCIF report

## Figures and Tables

**Table 1:** Hydrogen-bond geometry (Å, °)

*D*—H⋯*A*	*D*—H	H⋯*A*	*D*⋯*A*	*D*—H⋯*A*
O6—H11⋯O4^i^	0.82	2.01	2.826 (3)	172
O6—H12⋯O3^ii^	0.82	2.07	2.879 (3)	168
O5—H10⋯O6	0.82	2.02	2.830 (3)	168
O5—H9⋯O6^i^	0.82	2.11	2.871 (3)	155
O4—H8⋯O3^i^	0.82	1.90	2.725 (3)	176
O4—H7⋯O2^iii^	0.82	2.01	2.824 (3)	170
O1—H4⋯O2^i^	0.82	1.81	2.630 (2)	172
O1—H3⋯O5	0.82	1.88	2.697 (3)	174

Symmetry codes: (i) 
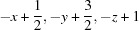
; (ii) 
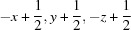
; (iii) 
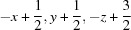
.

**Fig. 1.**
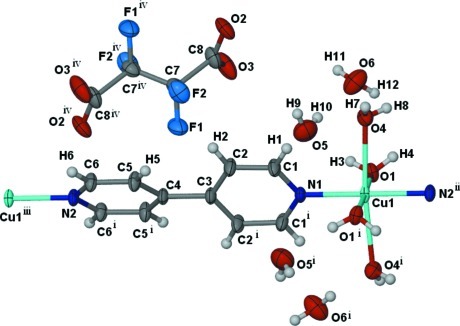
ORTEP drawing showing the coordination sphere of the Cu2+ center in the title compound with 50% probability displacement ellipsoids. Symmetry codes i: 1-x,y,1.5-z; ii: x,1+y,z; iii: x,1-y,z; iv: 0.5-z,0.5-y,1-z.

**Fig. 2.**
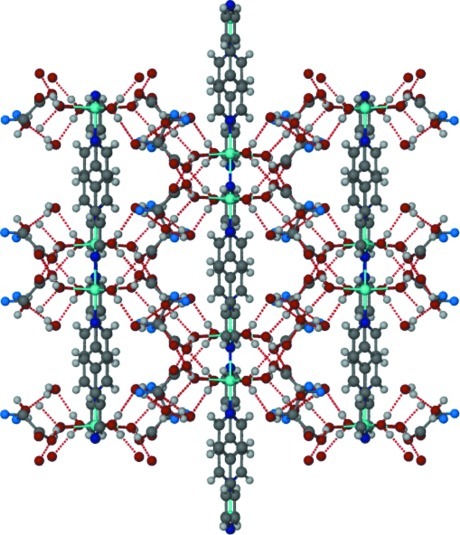
View down the c axis of the three-dimensional hydrogen bonding supramolecular network of the title compound.

## supplementary crystallographic information

### Comment

Metal-organic frameworks have been widely studied over the past few decades owing to their important applications in gas storage, catalysis, sensing, nonlinear optics, magnetism, luminescence and ferroelectricity (Allendorf *et al.*, 2009). Recently, the construction of hybrid framework materials using perfluorinated ligands has attracted much attention based on reports of interesting gas storage properties for such materials containing porous surfaces with exposed fluorine atoms (Yang *et al.*, 2007). Tetrafluorosuccinic acid, as a perfluorinated dicarboxylate ligand, is an excellent candidate for the construction of hybrid frameworks with diverse structures (Hulvey *et al.*, 2009) and with which the title compound, Cu(C_10_H_8_N_2_)(H_2_O)_4_.C_4_F_4_O_4_.4H_2_O, was hydrothermally prepared from Cu(NO_3_)_2_.3H_2_O and 4,4'-bipyridyl as coligand.

Both cation and anion have crystallographic 2-fold rotation symmetry with the 2-fold axes passing through Cu1, N1 and N2 and through the midpoint of the central C—C bond. The metal adopts a tetragonally elongated octahedral geometry because of the Jahn-Teller effect. The O4 atom occupies the elongated vertex with a Cu1—O4 distance of 2.462 (2) Å. The O1, N1 and N2 atoms occupy the equatorial plane with a Cu1—O1 distance of 1.976 (2) Å and Cu1—N1 and Cu1—N2 distances of 2.019 (3) and 2.027 (3) Å respectively (Figure 1). Adjacent Cu^II^ centers are bridged by 4,4'-bipy ligands to generate a one-dimensional linear chain structure parallel to the *b* axis. As shown in Figure 2 and Table 1, O—H···O hydrogen-bonding interactions between the cationic one-dimensional chains and the tetrafluorosuccinate anions and the free water molecules generate a three-dimensional supramolecular network.

### Experimental

A mixture of tetrafluorosuccinic acid (18.7 mg), 4,4'-bipyridyl (24.7 mg) and Cu(NO_3_)_2_.3H_2_O (15.2 mg) was dissolved in water (8 ml) and stirred for 0.5 h at room temperature. It was then sealed in a 25 ml Teflon-lined stainless steel reactor and heated at 393 K for 48 h. Blue crystals suitable for X-ray analysis were obtained after cooling the solution to room temperature. The yield is *ca* 70% based on Cu^2+^.

### Refinement

H atoms on O were located in difference maps and the O—H distances adjusted to 0.82 Å while H atoms on C were positioned geometrically with C—H = 0.93 Å. All were allowed to ride on their respective parent atoms with *U*_iso_(H) = 1.2 *U*eq(C or O).

### Crystal data

**Table:**

[Cu(C_10_H_8_N_2_)(H_2_O)_4_](C_4_F_4_O_4_)·4H_2_O	*F*(000) = 1132
*M**_r_* = 551.89	*D*_x_ = 1.641 Mg m^−^^3^
Monoclinic, *C*2/*c*	Mo *K*α radiation, λ = 0.71073 Å
Hall symbol: -C 2yc	Cell parameters from 8384 reflections
*a* = 17.112 (3) Å	θ = 3.0–27.4°
*b* = 11.135 (2) Å	µ = 1.07 mm^−^^1^
*c* = 12.126 (2) Å	*T* = 298 K
β = 104.85 (3)°	Block, blue
*V* = 2233.3 (7) Å^3^	0.44 × 0.22 × 0.10 mm
*Z* = 4	

### Data collection

**Table:**

Bruker SMART APEX diffractometer	2546 independent reflections
Radiation source: fine-focus sealed tube	2115 reflections with *I* > 2σ(*I*)
Graphite monochromator	*R*_int_ = 0.026
Detector resolution: 28 pixels mm^-1^	θ_max_ = 27.4°, θ_min_ = 3.0°
ω scans	*h* = −22→21
Absorption correction: multi-scan (*SADABS*; Bruker, 2005)	*k* = −14→14
*T*_min_ = 0.650, *T*_max_ = 0.900	*l* = −15→15
10662 measured reflections	

### Refinement

**Table:**

Refinement on *F*^2^	Secondary atom site location: difference Fourier map
Least-squares matrix: full	Hydrogen site location: inferred from neighbouring sites
*R*[*F*^2^ > 2σ(*F*^2^)] = 0.027	H-atom parameters constrained
*wR*(*F*^2^) = 0.085	*w* = 1/[σ^2^(*F*_o_^2^) + (0.0175*P*)^2^ + 4.9756*P*] where *P* = (*F*_o_^2^ + 2*F*_c_^2^)/3
*S* = 1.28	(Δ/σ)_max_ = 0.001
2546 reflections	Δρ_max_ = 0.70 e Å^−^^3^
153 parameters	Δρ_min_ = −0.78 e Å^−^^3^
0 restraints	Extinction correction: *SHELXL97* (Sheldrick, 2008), Fc^*^=kFc[1+0.001xFc^2^λ^3^/sin(2θ)]^-1/4^
Primary atom site location: structure-invariant direct methods	Extinction coefficient: 0.0077 (3)

### Special details

**Table:**

Geometry. All esds (except the esd in the dihedral angle between two l.s. planes) are estimated using the full covariance matrix. The cell esds are taken into account individually in the estimation of esds in distances, angles and torsion angles; correlations between esds in cell parameters are only used when they are defined by crystal symmetry. An approximate (isotropic) treatment of cell esds is used for estimating esds involving l.s. planes.
Refinement. Refinement of F^2^ against ALL reflections. The weighted R-factor wR and goodness of fit S are based on F^2^, conventional R-factors R are based on F, with F set to zero for negative F^2^. The threshold expression of F^2^ > 2sigma(F^2^) is used only for calculating R-factors(gt) etc. and is not relevant to the choice of reflections for refinement. R-factors based on F^2^ are statistically about twice as large as those based on F, and R- factors based on ALL data will be even larger.

### Fractional atomic coordinates and isotropic or equivalent isotropic displacement parameters (Å^2^)

**Table:**

	*x*	*y*	*z*	*U*_iso_*/*U*_eq_	
Cu1	0.5000	0.87438 (3)	0.7500	0.02173 (14)	
F1	0.33513 (10)	0.31057 (16)	0.46607 (16)	0.0488 (5)	
F2	0.29291 (11)	0.33959 (16)	0.61875 (14)	0.0457 (4)	
O1	0.43960 (11)	0.87146 (15)	0.58758 (13)	0.0277 (4)	
H3	0.4154	0.8089	0.5664	0.033*	
H4	0.4087	0.9285	0.5680	0.033*	
O2	0.16201 (13)	0.45430 (18)	0.49626 (16)	0.0426 (5)	
O3	0.20898 (16)	0.4317 (2)	0.34192 (18)	0.0559 (7)	
O4	0.36711 (11)	0.88591 (16)	0.79355 (15)	0.0307 (4)	
H7	0.3579	0.8968	0.8561	0.037*	
H8	0.3448	0.9399	0.7503	0.037*	
O5	0.35115 (14)	0.6752 (2)	0.5062 (2)	0.0533 (6)	
H9	0.3262	0.6610	0.5542	0.064*	
H10	0.3197	0.7005	0.4479	0.064*	
O6	0.23890 (14)	0.7923 (2)	0.32616 (18)	0.0540 (6)	
H12	0.2530	0.8407	0.2843	0.065*	
H11	0.2113	0.7397	0.2874	0.065*	
N1	0.5000	0.6931 (2)	0.7500	0.0212 (6)	
N2	0.5000	0.0563 (2)	0.7500	0.0231 (6)	
C1	0.45882 (16)	0.6311 (2)	0.8111 (2)	0.0296 (5)	
H1	0.4301	0.6731	0.8541	0.035*	
C2	0.45722 (17)	0.5067 (2)	0.8129 (2)	0.0315 (6)	
H2	0.4276	0.4668	0.8561	0.038*	
C3	0.5000	0.4417 (3)	0.7500	0.0244 (7)	
C4	0.5000	0.3076 (3)	0.7500	0.0246 (7)	
C5	0.48432 (17)	0.2427 (2)	0.8399 (2)	0.0298 (6)	
H5	0.4737	0.2827	0.9019	0.036*	
C6	0.48451 (16)	0.1187 (2)	0.8369 (2)	0.0277 (5)	
H6	0.4735	0.0766	0.8975	0.033*	
C7	0.26765 (16)	0.3133 (2)	0.5056 (2)	0.0339 (6)	
C8	0.20728 (18)	0.4103 (2)	0.4409 (2)	0.0363 (6)	

### Atomic displacement parameters (Å^2^)

**Table:**

	*U*^11^	*U*^22^	*U*^33^	*U*^12^	*U*^13^	*U*^23^
Cu1	0.0336 (3)	0.01019 (19)	0.0216 (2)	0.000	0.00738 (16)	0.000
F1	0.0408 (10)	0.0482 (10)	0.0676 (12)	0.0108 (8)	0.0323 (9)	0.0130 (9)
F2	0.0502 (10)	0.0491 (10)	0.0327 (8)	0.0104 (8)	0.0014 (7)	0.0004 (7)
O1	0.0379 (10)	0.0199 (8)	0.0241 (8)	0.0054 (7)	0.0058 (7)	0.0010 (7)
O2	0.0532 (13)	0.0414 (11)	0.0369 (10)	0.0262 (10)	0.0185 (9)	0.0090 (9)
O3	0.0837 (17)	0.0539 (14)	0.0366 (11)	0.0379 (13)	0.0276 (11)	0.0198 (10)
O4	0.0366 (10)	0.0288 (9)	0.0296 (9)	0.0033 (8)	0.0135 (7)	0.0058 (7)
O5	0.0492 (13)	0.0590 (14)	0.0498 (13)	−0.0107 (11)	0.0090 (10)	−0.0077 (11)
O6	0.0659 (15)	0.0598 (14)	0.0361 (11)	−0.0240 (12)	0.0125 (10)	−0.0009 (10)
N1	0.0260 (14)	0.0129 (12)	0.0245 (13)	0.000	0.0063 (11)	0.000
N2	0.0333 (16)	0.0125 (12)	0.0273 (14)	0.000	0.0145 (12)	0.000
C1	0.0395 (14)	0.0167 (11)	0.0392 (13)	0.0029 (10)	0.0224 (11)	−0.0002 (10)
C2	0.0433 (16)	0.0166 (11)	0.0427 (14)	−0.0001 (10)	0.0260 (12)	0.0033 (10)
C3	0.0317 (18)	0.0126 (14)	0.0307 (17)	0.000	0.0115 (14)	0.000
C4	0.0319 (18)	0.0121 (14)	0.0325 (17)	0.000	0.0130 (14)	0.000
C5	0.0484 (16)	0.0163 (11)	0.0303 (13)	−0.0009 (10)	0.0206 (11)	−0.0027 (9)
C6	0.0429 (15)	0.0178 (11)	0.0282 (12)	0.0002 (10)	0.0194 (11)	0.0016 (9)
C7	0.0338 (14)	0.0386 (15)	0.0323 (13)	0.0102 (12)	0.0140 (11)	0.0063 (11)
C8	0.0484 (17)	0.0291 (13)	0.0312 (13)	0.0147 (12)	0.0101 (12)	0.0082 (11)

### Geometric parameters (Å, º)

**Table:**

Cu1—O1^i^	1.9760 (17)	N1—C1	1.338 (3)
Cu1—O1	1.9761 (17)	N2—C6	1.344 (3)
Cu1—N1	2.018 (3)	N2—C6^i^	1.344 (3)
Cu1—N2^ii^	2.025 (3)	N2—Cu1^iii^	2.025 (3)
F1—C7	1.360 (3)	C1—C2	1.386 (3)
F2—C7	1.360 (3)	C1—H1	0.9300
O1—H3	0.8180	C2—C3	1.388 (3)
O1—H4	0.8212	C2—H2	0.9300
O2—C8	1.248 (3)	C3—C2^i^	1.388 (3)
O3—C8	1.232 (3)	C3—C4	1.494 (4)
O4—H7	0.8224	C4—C5	1.390 (3)
O4—H8	0.8241	C4—C5^i^	1.390 (3)
O5—H9	0.8196	C5—C6	1.382 (3)
O5—H10	0.8200	C5—H5	0.9300
O6—H12	0.8182	C6—H6	0.9300
O6—H11	0.8207	C7—C7^iv^	1.525 (6)
N1—C1^i^	1.338 (3)	C7—C8	1.560 (4)
			
O1^i^—Cu1—O1	178.11 (10)	C3—C2—H2	120.1
O1^i^—Cu1—N1	89.06 (5)	C2—C3—C2^i^	117.2 (3)
O1—Cu1—N1	89.06 (5)	C2—C3—C4	121.38 (15)
O1^i^—Cu1—N2^ii^	90.94 (5)	C2^i^—C3—C4	121.38 (15)
O1—Cu1—N2^ii^	90.94 (5)	C5—C4—C5^i^	117.4 (3)
N1—Cu1—N2^ii^	180.000 (1)	C5—C4—C3	121.31 (15)
Cu1—O1—H3	114.9	C5^i^—C4—C3	121.30 (15)
Cu1—O1—H4	114.1	C6—C5—C4	119.8 (2)
H3—O1—H4	109.3	C6—C5—H5	120.1
H7—O4—H8	108.2	C4—C5—H5	120.1
H9—O5—H10	109.4	N2—C6—C5	122.6 (2)
H12—O6—H11	109.5	N2—C6—H6	118.7
C1^i^—N1—C1	117.8 (3)	C5—C6—H6	118.7
C1^i^—N1—Cu1	121.09 (14)	F1—C7—F2	106.3 (2)
C1—N1—Cu1	121.09 (14)	F1—C7—C7^iv^	107.5 (3)
C6—N2—C6^i^	117.8 (3)	F2—C7—C7^iv^	107.8 (3)
C6—N2—Cu1^iii^	121.12 (14)	F1—C7—C8	110.5 (2)
C6^i^—N2—Cu1^iii^	121.12 (14)	F2—C7—C8	110.9 (2)
N1—C1—C2	122.7 (2)	C7^iv^—C7—C8	113.5 (3)
N1—C1—H1	118.7	O3—C8—O2	128.4 (3)
C2—C1—H1	118.7	O3—C8—C7	116.5 (2)
C1—C2—C3	119.8 (2)	O2—C8—C7	115.1 (2)
C1—C2—H2	120.1		

Symmetry codes: (i) −*x*+1, *y*, −*z*+3/2; (ii) *x*, *y*+1, *z*; (iii) *x*, *y*−1, *z*; (iv) −*x*+1/2, −*y*+1/2, −*z*+1.

### Hydrogen-bond geometry (Å, º)

**Table:**

*D*—H···*A*	*D*—H	H···*A*	*D*···*A*	*D*—H···*A*
O6—H11···O4^v^	0.82	2.01	2.826 (3)	172
O6—H12···O3^vi^	0.82	2.07	2.879 (3)	168
O5—H10···O6	0.82	2.02	2.830 (3)	168
O5—H9···O6^v^	0.82	2.11	2.871 (3)	155
O4—H8···O3^v^	0.82	1.90	2.725 (3)	176
O4—H7···O2^vii^	0.82	2.01	2.824 (3)	170
O1—H4···O2^v^	0.82	1.81	2.630 (2)	172
O1—H3···O5	0.82	1.88	2.697 (3)	174

Symmetry codes: (v) −*x*+1/2, −*y*+3/2, −*z*+1; (vi) −*x*+1/2, *y*+1/2, −*z*+1/2; (vii) −*x*+1/2, *y*+1/2, −*z*+3/2.
